# Association of Heterophil/Lymphocyte Ratio with Intestinal Barrier Function and Immune Response to *Salmonella enteritidis* Infection in Chicken

**DOI:** 10.3390/ani11123498

**Published:** 2021-12-08

**Authors:** Mamadou Thiam, Astrid Lissette Barreto Sánchez, Jin Zhang, Maiqing Zheng, Jie Wen, Guiping Zhao, Qiao Wang

**Affiliations:** State Key Laboratory of Animal Nutrition, Institute of Animal Sciences, Chinese Academy of Agricultural Sciences, Beijing 100193, China; mamadou.sleam@gmail.com (M.T.); asliss_07@hotmail.com (A.L.B.S.); zhangjin0913@126.com (J.Z.); zhengmaiqing@caas.cn (M.Z.); wenjie@caas.cn (J.W.); zhaoguiping@caas.cn (G.Z.)

**Keywords:** Chicken, H/L, Salmonella, goblet cells, mucosal morphology, intestine, gene expression

## Abstract

**Simple Summary:**

Salmonella represents a serious threat to the poultry industry and human health. The heterophil/lymphocyte (H/L) ratio indicates the robustness and immune system status of the chicken. Thus, the H/L ratio has been used for the selection of chickens that are resistant to Salmonella. However, the mechanisms conferring the resistance ability to the chickens with a low H/L ratio compared to those with a high H/L ratio remain unclear. Therefore, the present study aimed to investigate the association of the H/L ratio with the intestinal barrier function and immune response to *Salmonella enteritidis* infection in chicken. First, we enumerated the number of goblet cells in the ileum and caecum, measured the ileal villi morphology, and the expression of immune genes in the ileum and caecum of non-infected and SE-infected chickens at 7- and 21-days post-infection. Then, we assessed the correlation with the H/L ratio. The H/L ratio was negatively correlated to the number of goblet cells, IL-1β, IL-8, and IFN-γ ileal expressions, indicating that the individuals with a low H/L ratio displayed enhanced intestinal barrier and immunity. These results suggest that the H/L ratio is associated with intestinal immunity and could be a potential resistance indicator in chickens.

**Abstract:**

The heterophil/lymphocyte (H/L) ratio has been extensively studied to select poultry that are resistant to environmental stressors. Chickens with a low H/L ratio are superior to the chickens with a high H/L ratio in survival, immune response, and resistance to Salmonella infection. However, this disease resistance ability is likely to be associated with enhanced intestinal immunity. Therefore, to expand our understanding of these underlying resistance mechanisms, it is crucial to investigate the correlation between the H/L ratio as a blood immune indicator in live chickens and the intestinal barrier function and immunity. Jinxing yellow chickens H/L line one-day-old were divided into non-infected (NI) and *Salmonella enteritidis* infected (SI) at 7-days old. After dividing the birds into NI and SI, blood samples were taken for H/L ratios determination, and subsequently, birds from the SI group were infected with *Salmonella enteritidis* (SE). We assessed the effects of SE infection on the (i) goblet cells number from the ileum and caecum gut-segments, (ii) ileal mucosa morphology, and (iii) immune gene mRNA expressions from the ileum and caecum of NI and SI chickens at 7 and 21 days-post-infection (dpi). We found that the H/L ratio was negatively correlated with most intestinal immune indices, particularly with the goblet cells number and with IL-1β, IL-8, and IFN-γ ileal expressions. In conclusion, these results suggest that the H/L ratio is associated with the intestinal barrier and immune response for SE clearance and that the chickens with a low H/L ratio displayed enhanced intestinal immunity. This study expands the current knowledge that is related to using the H/L ratio to select and breed resistant broiler chickens.

## 1. Introduction

*Salmonella enteritidis* infection in chicken is a serious concern to the poultry business since is a source of contamination not only for other chickens in co-housing rearing facilities but also for the global human population via consumption of contaminated products [1]. Three possible illness outcomes are possible, depending on the host’s resistance and immune response competency, as well as the infecting serovars: acute/fatal Salmonellosis, chronic Salmonellosis, or bacterial clearance [2,3]. *Salmonella enteritidis* is one of the most common causes of food-borne disease [4,5]. The intestinal epithelium is the site of nutrient absorption and the first line of defense against several stressors that damage the intestinal epithelial barrier, resulting in immunological dysfunction and nutrient malabsorption in chickens [6,7,8]. *Salmonella* exposure impairs the function of the mucosal barrier and induces gut inflammation, which promotes *Salmonella* colonization in the chicken gut [9]. Therefore, intestinal mucosa integrity is vital for nutrient digestion and absorption, as well as general health.

One way to mitigate the danger of a Salmonella epidemic is by selecting and introducing resistant animals into breeding programs. Heterophils and lymphocytes are the two most abundant white blood cell types in birds, playing an essential role in innate and adaptative immunity, respectively [10]. While lymphocytes circulate through lymphoid tissues and the blood, they also establish residence in nonlymphoid organs, most notably in barrier tissues such as the intestines [11]. Therefore, the H/L ratio may represent a predisposition of resistance to infection by injury (via heterophils) rather than a communicable illness (via lymphocytes) [10]. Likewise, the H/L ratio in the blood reflects the immune system status [12]. It has also been used to select for responsiveness to the Newcastle disease vaccination, overall heat stress tolerance [13], and *Salmonella typhimurium* resistance [14]. Several studies reported this trait as highly heritable in poultry [14].

Preventing harmful bacterial invasion requires a well-developed intestinal epithelium and a completely intact mucosal barrier. Goblet cells release mucins, which are polysaccharide chains that are linked to a peptide backbone [15]. Pathogens are also prevented from entering epithelial cells by the mucus layer that is formed by mucin glycoproteins and water [16,17]. Salmonella infection changes the gut barrier and immune system by causing the production of cytokines such as interleukin-1 (IL-1β), interleukin-8 (IL-8), interleukin-6 (IL-6), lipopolysaccharide-induced tumor necrosis factor (LITAF), and interferon-(IFN-γ) [9,18,19,20,21]. However, the potential of chickens with a low H/L ratio to resist and clear Salmonella infection via the regulation of the intestinal inflammatory and immunological responses remains unclear.

The H/L ratio has been extensively studied for the selection of poultry that are resistant to environmental stressors [10,12,13,14,22]. However, to our knowledge, no studies have investigated the relationship between the H/L ratio and the intestinal immunity of the chicken in response to *Salmonella* infection. Therefore, the objectives of the present study were, firstly, to determine the effects of SE infection on goblet cells number, mucosal morphology, and immune-related genes expression in the ileum and caecum from NI and SI chickens. Secondly, this study aimed to assess the relationship between the H/L ratio that was measured at seven days-old and the intestinal barrier function and immunity of NI and SI chickens at 7 and 21 dpi. Thus, this study is potentially valuable for selecting Salmonella-resistant chickens and developing more specific disease-resistant chicken lines.

## 2. Materials and Methods

### 2.1. Ethics Approval, Animals and Experimental Design

The protocol for this study was reviewed and approved by the Institute of Animal Sciences’ Animal Welfare Committee (Chinese Academy of Agricultural Sciences, Beijing, China). Furthermore, animal experimentation and survival were approved by the IAS-CAAS Animal Ethics Committee (approval number: IAS2021-31).

In the present study, 210 Jinxing yellow chickens H/L line that were involved in a 21-days *Salmonella enteritidis* infection experiment were used for the associative analysis. Immediately after hatching, the chicks were transferred to housing rooms that were equipped with sterilized isolation ventilated cages (IVC) (IPQ-type 3 negative pressure isolator). The birds were randomly divided into 2 groups: NI and SI. After Salmonella infection, the birds of each group were assigned to one IVC with an average of 100 birds per cage. The temperature in the IVC was maintained at 37 °C the first week, and then was fixed at 35 °C with a weekly decrease of 2 °C until the experiment ended (21 dpi). The chicks received ad libitum specific-pathogen-free (SPF) feed (Beijing Keao Xieli Feed Co., Ltd., Beijing, China) and open access to sterilized water throughout the experiment.

### 2.2. Determination of the H/L Ratio

At 7 days old, 10 µL of fresh blood samples were collected from the basilic vein of each bird. The blood was taken and smeared on microscopic glass slides using a syringe, a needle, and a micropipette of 10 µL for drops of the same blood volume. The resulting blood smears were air-dried then stained using Giemsa staining. A total of 100 leukocytes were counted, including heterophils, lymphocytes, and monocytes, following a schematic diagram and using a Leica DM500 microscope with a magnification of 100× [23]. The H/L ratio was calculated by dividing the number of heterophile cells by lymphocyte cells. The descriptive statistics for monocytes, heterophils, lymphocytes cells, and H/L ratio are presented in Table 1.

### 2.3. Salmonella Infection and Sampling

*Salmonella enteritidis* 50335 (Institute of Veterinary drugs Control, Beijing, China) was used to infect the birds in this experiment. After resuscitation and growth of the bacteria at 37 °C in Luria Bertani broth (LB) with agitation (150 rpm) overnight, the concentrates were resuspended in sterile phosphate-buffered saline (PBS). The final number of colony-forming units (CFUs) was determined by plating in triplicate ten times serial dilutions on brilliant green agar (37 °C, overnight). Before infection, all of the chicks were checked for Salmonella presence by culturing cloacal swab samples in buffered peptone water overnight at 37 °C with agitation. According to the results, no infected chicks were detected. At 7-days-old, the birds from the SI group were infected by oral gavage with 1 mL of PBS containing 1 × 10^10^ CFUs of SE. The birds from the NI group received the same volume of sterile PBS.

At 7 and 21 dpi, 30 chickens from each experimental group were randomly selected and slaughtered. Then, the chickens were aseptically eviscerated and different gastrointestinal tract segment tissues (ileum and caecum) were aseptically sampled and stored in cryovial tubes at −80 °C for later mRNA expression analysis. In addition, tissues from the ileum and caecum gut segments were collected and stored in 4% paraformaldehyde for later histology analysis.

### 2.4. Ileum and Caecum Goblet Cells Count, and Ileal Villi Morphological Analysis

To assess the effects of SE infection on the intestinal barrier immune function indices at 7 and 21 dpi, 6 birds from NI and SI groups were used to analyze the goblet cells number [15] and the ileal villi morphometry [24]. First, segments of the ileum and caecum gut (2 cm length) were cut and washed three times with PBS to remove the intestinal contents. After abduction of the intestinal contents, tissues from each gut segment, time point post-infection, and bird were preserved in 2 mL sterile tubes with 4% paraformaldehyde for further histological analysis. Paraffin blocks of 3 mm of thickness were used to produce sections of 4 µm of thickness. The latest section was fixed on a microscopic glass slide and stained using Hematotoxin and Eosin (HE) and Alcian Blue-Passive Acidification Shift (AB-PAS, for goblet cells detection). For the determination of the goblet cells number (per villus or fold and crypt), ten villi (ileum), 5 folds (caecum), and 20 crypts that were representative and intact were used to count the number of goblet cells in each indicated intestinal structure. Concerning the morphometry analysis of the ileal villi, ten representative intact villi and their associated crypt were selected to measure the villus height (VH), crypt depth (CD), villus height/crypt depth ratio (VH/CD), villus width (VW), villus surface area (VSA = VH × VW × π), epithelium thickness (EPT), and Lamina propria thickness (LPT) using Image J (Wayne Rasband and contributors, National Institutes of Health USA). The pictures were captured using a light microscope Leica DMI6000B (Wetzlar, Germany) that was equipped with Leica Application Suite (LAS) image-processing software.

### 2.5. RNA Extraction, cDNA Synthesis, and Quantitative Real-Time PCR (RT-QPCR)

RNA extraction from the ileum and caecum tissues was carried out with TRIzol reagent (Thermo Fisher Scientific, Waltham, MA, USA) as described by the manufacturer. Total RNA was quantified using a NanoDrop spectrophotometer (ND1000; Thermo Fisher Scientific Inc., Waltham, MA, USA). The cDNA synthesis was performed using one microgram of RNA reverse transcribed using a FastKing gDNA Dispelling RT SuperMix cDNA synthesis kit (Tiangen, Beijing, China), which was used following the manufacturer’s instructions. For quantitative PCR, cDNA was diluted 1:5 in RNase- and DNase-free water. The expression of IL-1β, IL-6, IL-8, IFN-γ, LITAF, SOCS3, Muc2, Claudin-1, NF-κB, and TLR4 were carried out from cDNA of Ileum and caecum tissues, using specific primers. The mRNA expression was performed using the Applied Biosystems SYBR GREN master mix (Thermo Fisher Scientific, Vilnius, Lithuania). The amplification system was as follows: 1.5 µL of cDNA (10-fold diluted), 5 µL of SYBR GRENN master mix, 0.4 µL of forward and reverse primers, and adding double-distilled water up to final reaction volume (10 µL). The assays were performed using ABI 7500 (Applied Biosystems, Forester City, CA, USA). The Q-PCR run method was 95 °C for 3 min, 40 cycles of 95 °C 3 s, and 60 °C for 34 s. First, the expression of each targeted gene was normalized by the housekeeping genes GAPDH. Then, the abundance of each target mRNA was assessed by a 2^−∆∆CT^ comparative method [25], as the following formula:

−ΔΔCT (sample − control) = (CT of target gene − CT of GAPDH gene) _sample_ − (CT of target gene − CT of GAPDH gene) _control_. The primers that were used in this study are listed in Table 2.

### 2.6. Statistical Analysis

The results are expressed as the mean and standard error of the mean (SEM). GraphPad Prism version 8 (GraphPad Software, San Diego, CA, USA) and R version 4.0 were used to analyze the data. Differences in the villus morphometry between the two groups were analyzed using Student’s *t*-test (one Tail) at each time point post-infection. The changes in goblet cells and mRNA expression at 7 and 21-dpi in non-infected and SE-infected chickens were analyzed using two-way ANOVA with Sidak’s multiple comparisons in GraphPad Prism. Correlations between 2 factors were performed using Pearson’s correlation in GraphPad Prism, with the significance determined by Student *t*-test (one Tail). Correlogram of Pearson’s correlation between H/L and the different villus morphological indices were performed using ggcorrplot and corrplot R packages. Statistical significance was declared when the *p*-value was < 0.05.

## 3. Results

### 3.1. Salmonella enteritidis Infection Decreases the Number of Goblet Cells

**Figure 1:**
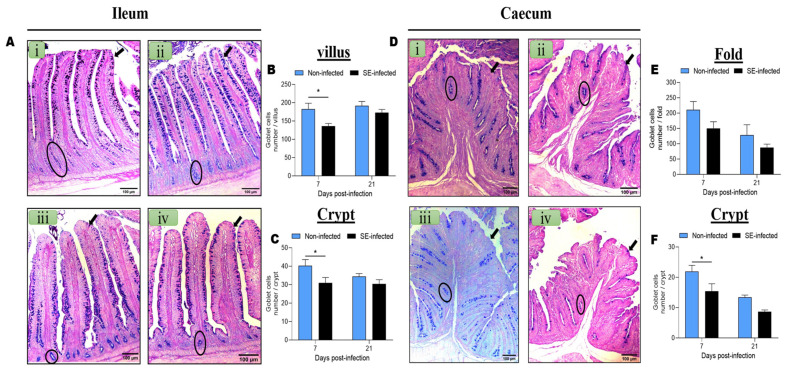
Effects of *Salmonella enteritidis* infection on the ileal and cecal goblet cells number at 7 and 21-days post-infection. (**A**) Ileum villi and crypt of non-infected and SE-infected chickens at 7 and 21 dpi (i = 7 dpi NI, ii = 7 dpi SI, iii = 21 dpi NI and iv = 21 dpi SI); the arrow is indicating the villus and the circle the crypt. (**B**) Ileum goblet cells number in the villi at 7 and 21 dpi (n = 6). (**C**) Ileum goblet cells number in the crypts at 7 and 21 dpi (n = 6). (**D**) Caecum folds and crypts of non-infected and SE-infected chickens at 7 and 21 dpi the arrow indicating the fold and the circle the crypt (i = 7 dpi NI, ii = 7 dpi SI, iii = 21 dpi NI and iv = 21 dpi SI). (**E**) Caecum goblet cells number in the folds at 7 and 21 dpi (n = 5). (**F**) Caecum goblet cells number in the crypts at 7 and 21 dpi (n = 5). Data were analyzed by 2-way ANOVA with Sidak’s post-test α = 0.05; * *p* < 0.05. Images were captured at 100× magnification.

To evaluate the effects of SE infection on the goblet cell dynamics (number per villi, folds, and crypts) from the ileum and caecum of NI and SI chickens at 7 and 21 dpi, we enumerated the number of goblet cells. Figure 1A presents the distribution of the goblet cells along the ileal villi and crypts of NI and SI chickens at 7 and 21 dpi. The distribution of the goblet cells along the folds and crypts in the caecum of NI and SI chickens at 7 and 21 dpi are presented in Figure 1D. The distribution of the goblet cells in the ileum and caecum of NI and SI chickens at 7 and 21 dpi showed that the number of goblet cells in the caecum decreased with the time under both conditions, while in the ileum, the number of goblet cells remained approximatively constant across the time for both groups of chickens. The enumeration of the goblet cells along the ileal villi and crypts of the two groups at 7 and 21 dpi showed that SE infection significantly decreased (*p* < 0.05) the number of goblet cells in the villi (Figure 1B) and crypts (Figure 1C) at 7 dpi. In the caecum, SE infection significantly decreased (*p* < 0.05) the number of goblet cells only in the crypt (Figure 1F) at 7 dpi.

### 3.2. Association of H/L Ratio with the Number of Goblet Cells in the Ileum and Caecum

The association of the H/L ratio that was measured at 7-days-old with the goblet cells number in the ileum and caecum of NI and SI chickens at 7 and 21 dpi are summarized in Table 3. It was remarkable that the H/L ratio was negatively correlated with the number of goblet cells in the ileum and caecum of chickens from the two groups. Interestingly, Pearson’s correlation analysis showed that the H/L ratio was significantly and negatively correlated with the number of goblet cells in the villi and crypts of NI chickens only at 21 dpi.

### 3.3. Effects of Salmonella enteritidis Infection on the Ileal Villi Integrity

**Figure 2:**
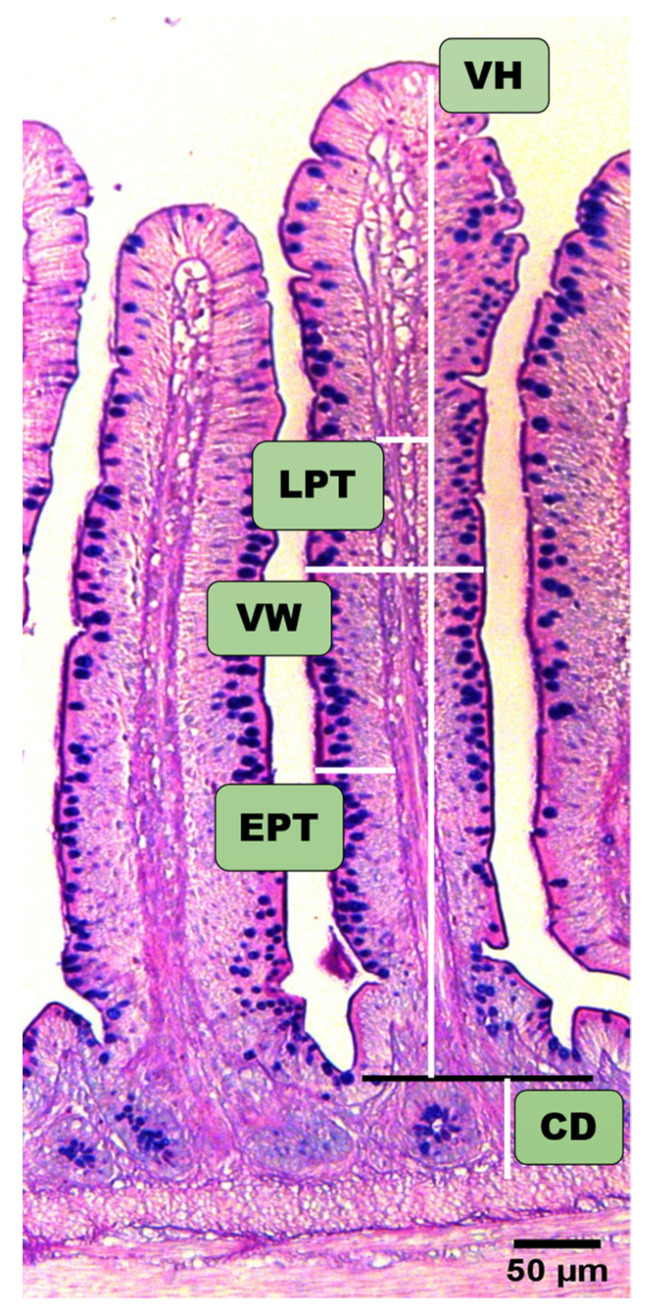
Description of the ileal villi morphology indices. At 7 and 21 dpi, sections of 5µm of thickness from the ileum stained using HE and AB-PAS were used for intestinal villi morphometry (n = 5). Villus height (VH), crypt depth (CD), villus height/crypt depth ratio (VH/CD), villus width (VW), villus surface area (VSA) = π × villus height × villus width, epithelium thickness (EPT), and lamina propria thickness (LPT).

To investigate the effects of time and SE infection on the ileal villi histomorphometry, villus height (VH), crypt depth (CD), villus height/crypt depth ratio (VH/CD), villus width (VW), villus surface area (VSA) = π × villus height × villus width, epithelium thickness (EPT), and lamina propria thickness (LPT) were measured (Figure 2). Table 4 presents the effects of SE infection on the ileal villi morphometry at 7 and 21 dpi. At 7 dpi, our results showed that the SE infection did not affect the CD, VH/CD, and LPT. However, we observed that SI chickens displayed significantly decreased VH, VW, VSA, and EPT than NI chickens. At 21 dpi, we found that SE infection significantly reduced the VH, VSA, and VH/CD but significantly increased the LPT. It was noteworthy that despite the absence of a significant difference between the NI and SI chickens, SE infection increased the VW and EPT on day 21 post-infection.

### 3.4. Association between H/L Ratio and Important Ileal Villi Morphometry Parameters

**Figure 3:**
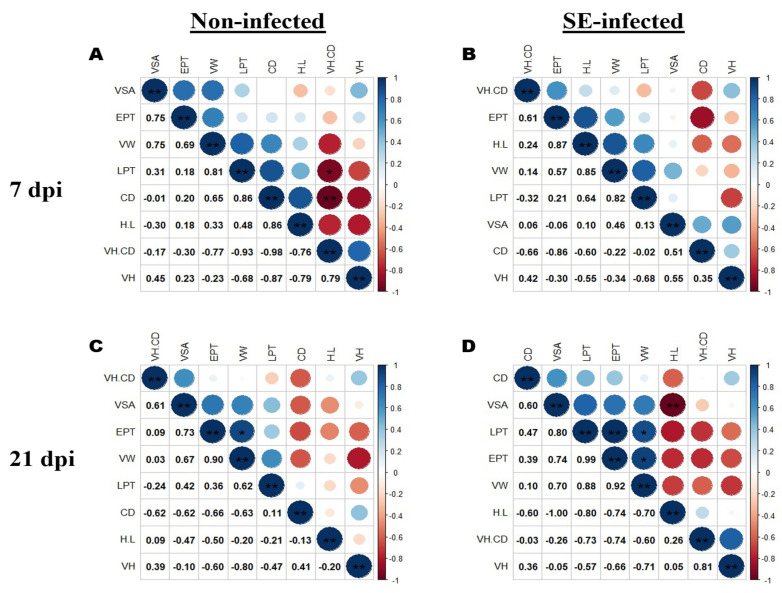
Association among the H/L ratio and the ileal villi morphometry indices (n = 5). (**A**) Pearson correlation among different parameters recorded from non-infected chickens at 7 dpi. (**B**) Pearson correlation among different parameters recorded from SE-infected chickens at 7 dpi. (**C**) Pearson correlation among different parameters recorded from non-infected chickens at 21 dpi. (**D**) Pearson correlation among different parameters recorded from SE-infected chickens at 21 dpi. The blue color indicates positive correlations and the red color indicates negative correlations. H/L ratio (H.L), Villus height (VH), crypt depth (CD), villus height/crypt depth ratio (VH.CD), villus width (VW), villus surface area (VSA) = π × villus height × villus width, epithelium thickness (EPT), lamina propria thickness (LPT). * *p* < 0.05, ** *p* < 0.01.

To assess the relationship between the H/L ratio and the different ileal villi morphology indices, we performed a series of Pearson’s correlation analyses, illustrated by correlograms that are representative of each chickens’ group and time point post-infection. Figure 3 summarizes the correlations among H/L, VH, CD, VW, VSA, VH/CD, EPT, and LPT from NI and SI chickens at 7 and 21-dpi. From this Figure, those parameters are correlated under normal conditions, and SE infection negatively affected their relationship across time. The H/L ratio was associated with important histomorphometric parameters and a low H/L ratio was correlated with increased VSA and CD under Salmonella infection at 21 dpi (Figure 3D).

### 3.5. Effects of Salmonella Infection on the Expression of Immune Response-Related Genes

**Figure 4:**
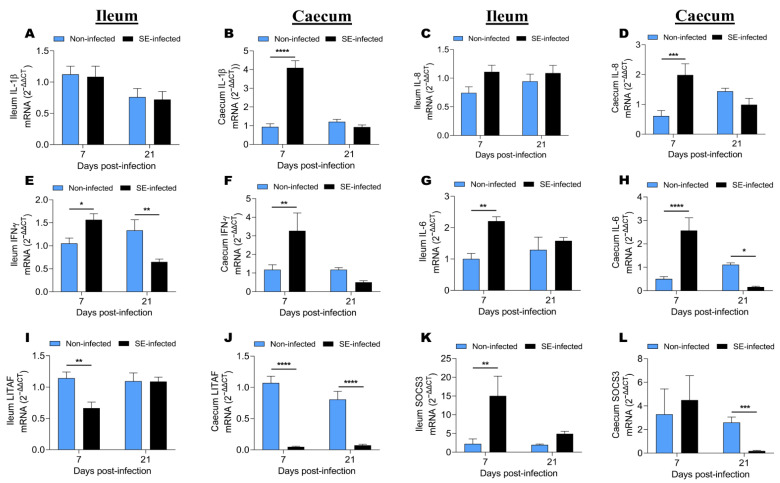
Effects of SE infection on the expression of genes related to the intestinal immune response at 7 and 21 dpi. (**A**,**B**) Interleukin-1β (IL-1β) mRNA expression in the ileum and caecum, respectively. (**C**,**D**) Interleukin-8 (IL-8) mRNA expression in the ileum and caecum, respectively. (**E**,**F**) Interferon-γ (IFN-γ) mRNA expression in the ileum and caecum, respectively. (**G**,**H**) Interleukin-6 (IL-6) mRNA expression in the ileum and caecum, respectively. (**I**,**J**) Liposaccharide-induced tumor necrosis factor (LITAF) mRNA expression in the ileum and caecum, respectively. (**K**,**L**) Suppressor of cytokine signaling 3 (SOCS3) mRNA expression in the ileum and caecum, respectively. The assay included 9 to 10 birds per group at each time point, except for SOCS3, where the assay included 6 birds. Data were analyzed by 2-way ANOVA with Sidak’s post-test α = 0.05; * *p* < 0.05, ** *p* < 0.01, *** *p* < 0.001, **** *p* < 0.0001.

To determine the effects of SE infection on the regulation of genes that are related to the intestinal immune response, we analyzed the mRNA expression of IL-1β, IL-8, IFN-γ, IL-6, LITAF, and SOCS3 in the ileum and caecum of NI and SI chickens at 7 and 21 dpi (Figure 4). The expression of IL-1β, IL-8, and IFN-γ were up-regulated in the caecum (Figure 4B,D,F) compared to that of the ileum (Figure 4A,C,E). However, the expression of SOCS3 was up-regulated in the ileum (Figure 4K) compared to that of the caecum (Figure 4L), while the expression of IL-6 and LITAF remained approximatively similar in the ileum and caecum (Figure 4G–J). SE did not affect the expression of IL-1β and IL-8 in the ileum (Figure 4A,C), while in the caecum, the SE infection significantly increased IL-1β and IL-8 expression (Figure 4B,D) at 7 dpi (*p* < 0.0001 and *p* < 0.001, respectively). In the ileum, SE infection increased the expression of IFN-γ, IL-6, and SOCS3 (Figure 4E,G,K) at 7 and 21 dpi (excepted IFN-γ expression at 21 dpi; Figure 4E, which was significantly (*p* < 0.01) decreased). In the caecum, *Salmonella enteritidis* infection increased the expression of IFN-γ, IL-6, and SOCS3 at 7 dpi, while at 21 dpi the expression of IL-6 and SOCS3 decreased significantly (*p* < 0.05 and *p* < 0.001 respectively) (Figure 4F,H,L). Remarkably, we found that in the caecum, SE infection significantly (*p* < 0.0001) decreased the expression of LITAF at 7 and 21 dpi (Figure 4J).

### 3.6. Effects of Salmonella Infection on the Expression of Genes Related to Intestinal Barrier Functions

**Figure 5:**
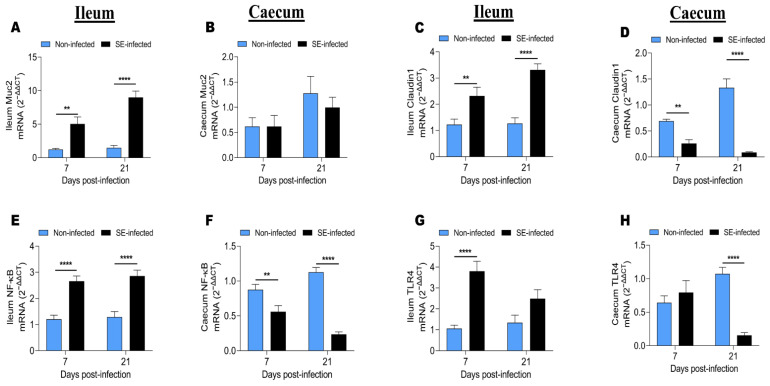
Effects of SE infection on the expression of genes related to the intestinal immune barrier at 7 and 21 dpi. (**A**,**B**) Mucin 2 (Muc2) mRNA expression in the ileum and caecum, respectively. (**C**,**D**) Claudin1 mRNA expression in the ileum and caecum, respectively. (**E**,**F**) Nuclear factor-κB (NF-κB) mRNA expression in the ileum and caecum, respectively. (**G**,**H**) Toll-like receptor 4 (TLR4) mRNA expression in the ileum and caecum, respectively. The assay included 9 to 10 birds per group at each time point. Data were analyzed by 2-way ANOVA with Sidak’s post-test α = 0.05; ** *p* < 0.01, **** *p* < 0.0001.

To investigate the effects of SE infection on the expression of genes related to the intestinal immune barrier function, we determined the expression of Muc2, Claudin-1, NF-κB, and TLR4 from the ileum and caecum of NI and SI chickens at 7 and 21 dpi (Figure 5). The results showed that the expression of those genes was up-regulated in the ileum compared to that of the caecum. Moreover, we found that the expression of Muc2, Claudin-1, NF-κB, and TLR4 in the ileum at 7 and 21-dpi from the chickens of the two groups (Figure 5A,C,E,G) were inversely regulated, compared to that of the caecum (Figure 5B,D,F,H). In the ileum, SE infection significantly increased the expression of Muc2, Claudin-1, NF-κB, and TLR4 at 7 and 21 dpi (except for TLR4 expression at 21 dpi, which was increased but without statistical significance). In the caecum, the SE infection did not affect the expression of Muc2 at 7 and 21 dpi. However, in the same gut segment, SE significantly decreased the expression of Claudin-1, NF-κB, and TLR4 (except for TLR4 expression at 7 dpi, which was increased without statistical significance).

### 3.7. Correlation between H/L Ratio and Intestinal Gene Expression

To find out with which genes related to the intestinal barrier function and immunity the H/L ratio is associated, we performed a series of Pearson’s correlation tests between the H/L ratio that was measured at 7-days-old and the mRNA expression level of IL-1β, IL-8, IFN-γ, IL-6, LITAF, SOCS3, Muc2, Claudin-1, NF-κB, and TLR4 from the ileum and caecum of NI and SI chickens at 7 and 21 dpi (Table 5). Under both conditions and at the 2-time points post-infection, we found more negative than positive correlations indicating that a low H/L ratio was associated with increased expression of those genes. No significant correlations of the H/L ratio with Claudin-1 and TLR4 were found. The H/L ratio was correlated to IL-1β and IL-8 expressions only from the ileum of SI chickens at 7 and 21 dpi. We remarked that the expression of those two genes were significantly and negatively correlated (r = −0.62, *p* < 0.05 and r = −0.58, *p* < 0.05, respectively) to the H/L ratio at 7 dpi, while at 21 dpi they were significantly and positively correlated (r = 0.68, *p* < 0.05 and r = 0.70, *p* < 0.05, respectively) to the H/L ratio. IFN-γ mRNA expression was correlated to the H/L ratio only at 7 dpi and from the ileum of SI chickens (r = −0.67, *p* < 0.05). Interestingly, the results showed that IL-6 expression was positively correlated to the H/L ratio from the ileum of NI and SE at 21 dpi (r = 0.63, *p* < 0.05 and r = 0.64, *p* < 0.05, respectively); and from the caecum of NI at 7 dpi (r = −0.51, *p* < 0.05). LITAF expression was correlated to the H/L ratio from the ileum of NI chickens at 7 and 21 dpi (r = 0.58, *p* < 0.05 and r = −0.80, *p* < 0.01, respectively), while SOCS3 expression was correlated to the H/L ratio only from the caecum of NI chickens at 21 dpi (r = −0.68, *p* < 0.01). Muc2 and NF-κB expression from the caecum of NI chickens were correlated to the H/L ratio only at 7 dpi (r = −0.63, *p* < 0.05 and r = −0.59, *p* < 0.05, respectively).

## 4. Discussion

The present study assessed the effects of SE on important intestinal barrier function and immunity parameters and established the relationship between those parameters and the H/L ratio. The intestinal epithelium is the major location of nutrient absorption and the first line of defense against many stimuli that have been found to damage the intestinal epithelial barrier, resulting in immunological dysfunction and nutrient loss in chickens [6,7,8,24]. The intestine of birds is involved in fermentation, transport, and nutritional absorption. Apart from its food absorption and transport activities, the intestinal epithelium also acts as a barrier against pathogenic microorganisms and hazardous chemicals [28]. Chronic *Salmonella* infection can damage the gut mucosal barrier and disrupt intestinal homeostasis [29]. Therefore, *Salmonella* reduction in chickens’ intestines needs an understanding of the interactions between *Salmonella* and the intestinal immune components that serve as the first line of defense. The mucus layer (mucins produced by goblet cells) and the underlying epithelial barriers are examples of such features [30]. Goblet cells are generated from pluripotent stem cells found in the intestinal crypt and are essential in the development and absorption of intestinal epithelial cells [31,32]. Glycoproteins that are produced by the goblet cells effectively flush external pathogenic agents and prevent harmful chemicals from contacting the intestinal epithelial cells [33,34]. The present study showed that SE significantly decreased the number of goblet cells in the ileum and caecum of infected chickens, following the previous studies [28,35,36,37]. Although some researchers showed that *Salmonella* infection increases the goblet cells density [30], we observed a depletion of goblet cells in the present work. Moreover, we illustrated that the number of goblet cells in the caecum decreased significantly from 7 to 21 dpi under normal and infected conditions.

As the chicks develop, they naturally acquire a protective microflora in their intestines, increasing their resistance to *Salmonella* and other enteric infections [38,39]. Intestinal bacteria may influence goblet cell development and the mucus layer directly or indirectly via the activation of host immune cells [40]. Therefore, we also assessed the relationship between the H/L ratio and the number of goblet cells in the ileum and caecum gut segments. We found that the H/L ratio was negatively correlated to the number of goblet cells, indicating that chickens with a low H/L ratio displayed increased goblet cells number. This result may be explained by the high number of lymphocyte cells, characteristic of a low H/L ratio. T lymphocytes can defend the host by removing infected cells or recruiting other immuno-protective and immune regulatory cells [11].

Many histopathologic alterations occur when a pathogen breaks the mucus barrier, colonizes, and invades the intestinal epithelium [30]. The main symptoms of Salmonellosis occur during the first 7-days post-infection. Salmonella destroys and invades the intestinal epithelial tissue during this period and the host immune system fights to protect and clear this pathogen [41]. The present results showed that SE infection significantly decreased the VH (at 7 and 21 dpi), VW (at 7 dpi), VSA (at 7 and 21 dpi), VH/CD (at 21 dpi), and EPT (at 7 dpi). Our results are consistent with the previous studies that reported that *Salmonella* infection in chickens decreases the villus height, crypt depth, villus surface area, and villus height/crypt depth ratio [24,28,30,42,43]. Moreover, the current work found that *Salmonella* infection significantly increased the LPT (at 21 dpi), increasing inflammatory cell infiltration. Fasina et al. [30] reported a substantial increase of the LPT in two studies that were evaluating the influence of *Salmonella typhimurium* (ST) infection on intestinal goblet cell dynamics (density and size) and villous shape in broiler chicks [44,45,46,47]. Investigating the correlation between the H/L ratio and the important ileal villi morphology parameters showed that those parameters are correlated under normal conditions, and SE infection affects the link between them at 7 and 21 dpi. At 21 dpi and under Salmonella infection, the H/L ratio was significantly and negatively correlated with villus surface area (VSA) and crypt depth (CD), indicating that under Salmonella infection a low H/L ratio is associated with increased VSA and CD. An increased VSA is associated with high capability of absorbing available nutrients [48], while an increase in CD is associated with fast tissue turnover which is necessary for villus renewal [49]. From the analysis of all correlograms, we deducted that the H/L ratio is correlated to the intestinal villi morphology indices, and the chickens with a low H/L ratio display improved intestinal integrity compared to that of the chickens with a high H/L ratio at the earlier (7 dpi) and later (21 dpi) stage post-SE infection.

Cytokines play a central role in immunity [50]. They are innate and adaptive immune system effector messengers that start and regulate immune responses to eliminate infections [51]. Chemokines are a subclass of cytokines with chemoattractant properties that control the migration of immune cells [52]. Salmonella infection is characterized by the induction of an inflammatory response and a change in the expression of particular genes. The changes can be an upregulation or downregulation in the function of the immune system status of the host.

This study compared the expression of proinflammatory cytokines, chemokines, and essential signaling pathways genes from the ileum and caecum between NI and SI chickens. The results showed that the expression of IL-1β, IL-8, IFN-γ, and SOCS3 genes in the ileum was different from that of the caecum of the chickens, while IL-6 and LITAF expressions remained approximatively similar in these two gut segments. Fasina et al. [53] observed a significant change in cytokines expression only from the ileum gut segments, with the leading cause the presence of Peyer’s patch in the ileum and the absence of the cecal tonsils (the primary lymphoid nodule) from the caecum gut segment [54]. In contrast, the present results showed significant changes in the ileum and caecum of SE chickens compared to the NI group. This difference with our results may be due to the age of the chickens that were used in our experiment. Knowing that the development of the resistance to Salmonella infection in chickens is positively correlated with age; it has been demonstrated that young chicks are more susceptible to Salmonella infection than older chickens [38]. Chickens become increasingly resistant to Salmonella infection as they mature due to the development of their gastrointestinal and immune systems [55,56]. Our results also showed that in the ileum, SE did not affect the expression of IL-1β and IL-8, compared to those of the caecum, which were significantly increased at 7 dpi. Consistent with our results, Kaiser et al. [57] discovered that *Salmonella gallinarum* invasion of avian cells did not generate any inflammatory cytokines (IL-1β or IL-6), whereas SE colonization resulted in the downregulation of IL-1β. Withanage et al. [58] observed no change in intestinal IL-1β expression between 1 and 7 dpi in a seven day-old SPF Rhode Island Red chicks that were infected with 10^8^ CFU of ST. Accordingly, we did not find a significant difference between NI and SI chickens’ IL-1β expression from the ileum. However, from the caecum, we observed a substantial difference between these two groups at 7 dpi. This difference may be due to the breed (Jinxing yellow chickens H/L line), Salmonella type that was used (SE), and the dosage (10^10^ CFU).

In contrast, other studies reported an increase in IL-1β and IL-8 during the days following Salmonella infection [59,60]. For example, Quinteiro-Filho et al. [61] reported an increase in IL-1β expression from the cecal tonsils of SE-infected chickens [46,62,63]. Furthermore, IL-1β inoculation decreases Salmonella-infected mice’s mortality [64], while the neutralization or inhibition of IL-1β production increases the severity of Salmonella infection, reducing the host survival rate [62].

IFN-γ, that is produced by T lymphocytes and natural killer cells, is a pleitropic chemical that has a distinct effect on each step of the immune response [65,66,67,68]. The current study observed that SE infection increased IFN-γ, IL-6, and SOCS3 in the chickens’ ileum and caecum, particularly at 7 dpi [9,59,60]. In accordance with our observations, Dar et al. [50], in an experiment including chickens that were artificially infected by ST for 15 days, reported an increase in IFN-γ with a pick at 5 dpi from the caecum and spleen. Moreover, Fasina et al. [53] reported an increase in IFN-γ expression from the ileum at 10 dpi following ST infection, while Whithanage et al. [58] observed upregulation in IFN-γ expression from the same gut-segment from 3 to 14 dpi in SPF Rhode Island Red chicks that were infected with 10^8^ CFU of ST at 7 day-olds.

LITAF and IL-6 are pro-inflammatory cytokines that play essential roles in intestinal inflammation [69]. LITAF is expressed in all chicken leukocytes and lymphocytes subpopulations, whereas IL-6 is primarily produced by chicken macrophages [60]. Furthermore, following infectious disease, LITAF, IL-6, and IFN-γ production can impair the intestinal tight junction barrier [69]. In the present work, SE infection up-regulated LITAF expression in the ileum, while in the caecum, it significantly decreased its expression at 7 and 21 dpi. Our results are consistent with those of Song et al. [9], who observed that SE infection induces an increase in mucosal genes expressions, such as IL-1b, IL-6, IFN-γ, SOCS3, and LITAF.

Muc2 and Claudin-1 are vital intestinal epithelial barrier components that prevent the intestinal epithelial cells from reaching harmful substances or pathogens [24]. The mucus that is produced by the intestinal goblet cells acts as an important barrier against ST invasion [35,70]. A dense mucus layer covers the ileum and colon, but the caecum has a patchy mucus layer and so the cecal epithelium is the major entrance route for ST in mice [71]. Mucus composition and glycosylation patterns affect the structure of the bacterial population and susceptibility to *Salmonella* infection [72]. The current study showed that the Muc2, Claudin-1, NF-κB, and TLR4 expression were up-regulated in the ileum, compared to the caecum of chickens from NI and SI groups at 7 and 21 dpi. Claudin-1 is one of the crucial tight junction proteins; higher epithelial tightness and lower solute permeability are associated with increased Claudin-1 expression [73]. Heat stress and bacterial pathogens can affect this permeability [74,75,76]. In the ileum, SE infection increased Muc2, Claudin-1, NF-κB, and TLR4 (only at 7 dpi) expression at 7 and 21 dpi. However, the results showed that SE did not affect Muc2 expression at 7 and 21 dpi in the caecum, while it significantly decreased Claudin1, NF-κB, and TLR4 (excepted at 7 dpi) gene expressions at 7 and 21 dpi.

In contrast with the present study results, Song et al. [9] observed a decrease in Muc2 and Claudin-1 gene expression levels from the ileum SE-infected chickens. However, previous studies also reported an increase in TLR4 mRNA expression that was induced by Salmonella infection [9,77,78]. Increased TLR4 expression caused by SE exposure is critical for controlling intestinal mucosal immune responses, barrier function, and proinflammatory cytokine production [77,78].

Chicken heterophils are the most common leukocyte population in the caecal lamina propria without infection, followed by macrophages and T-cells [60]. However, after Salmonella infection, the increase in heterophils’ population is the lowest compared to the other leukocyte subpopulations. Globally, the current work showed that under normal and infected conditions, the H/L ratio was negatively correlated to most genes related to the intestinal barrier and immune response, suggesting that the chickens with a low H/L ratio displayed enhanced intestinal immunity. These results follow the findings of Al-Murrani et al. [13] and Genovese et al. [79] which have reported that the chickens and offspring of chickens with a low H/L ratio are superior to the chickens with a high H/L ratio in terms of productivity, adaptability, Salmonella resistance, and immune response. However, our data revealed that the H/L ratio was not correlated with Claudin-1 and TLR4 expression.

IL-1β and IL-8 expressions from the ileum of SI chickens were negatively correlated to the H/L ratio on day seven and positively correlated to the H/L ratio on day 21 post-infection. Kogut, Rothwell, and Kaiser [59] reported that chickens heterophils express IL-8 after receptor-mediated phagocytosis; this was consistent with our study where we observed that IL-8 expression from the ileum of SI chickens was positively correlated to the H/L ratio at 21 dpi, suggesting that a high H/L ratio, which is characteristic of increased heterophils, enhanced IL-8 expression. Heterophils play a crucial role in the early prevention of SE infection in chickens by rapidly migrating to the invasion site [45,47,80].

This study found that the H/L ratio was negatively correlated with IFN-γ expression from the ileum of SI chickens at 7 dpi, indicating that a low H/L ratio is associated with enhanced IFN-γ ileal expression under Salmonella infection. Several previous studies have demonstrated that the rate of Salmonella infection clearance corresponds with an increase in IFN-γ mRNA expression and a robust T-cell response [46,55]. Accordingly, our study observed that SE-infected chickens with a low H/L ratio, characteristic of increased peripheral blood lymphocyte cells, displayed increased IFN-γ mRNA expression from the ileum at 7 dpi. These results suggest that a low H/L ratio it’s an immunological status that can provide earlier and rapid clearance of Salmonella infection in chicken. Furthermore, it has been proven that ST infection clearance is related to a high level of cell-mediated responses associated with a robust T-cell response, not with a high level of antibody [81,82]. Therefore, the correlation found between the H/L ratio and the IFN-γ may be explained by the predisposition of chickens with a low H/L ratio to display enhanced T-cell response compared to chickens with a high H/L ratio. IFN-γ has been found to play a critical role in the clearance of intestinal Salmonella infections [83]. However, it has been demonstrated that IFN-γ production that is caused by Salmonella infection impairs the homeostasis of the gut mucosa, including the microbial population, epithelial cells, and the gut immune system [84]. In addition, the current study also showed that IL-6 expression was positively correlated to the H/L ratio from the ileum of NI and SI chickens at 21 dpi; LITAF expression from the ileum of NI chickens was positively and negatively correlated to the H/L ratio at 7 and 21 dpi, respectively; a negative correlation between the H/L ratio and SOCS3 expression from the caecum of NI Chickens was found; and Muc2 and NF-κB expression from the caecum of NI chickens on day seven post-infection were negatively correlated to the H/L ratio.

In this work, we observed a strong correlation of the H/L ratio with IL-1β and IFN-γ intestinal expression, indicating that the H/L ratio can be used as a blood indicator to select and predict the Salmonella infection resistance also to predict the intestinal immunity level in vivo in chickens. Accordingly, Fasina et al. [53] concluded that IL-1β and IFN-γ are critical elements to consider when developing immunotherapeutic preventive strategies (such as vaccinations or cytokines treatment) for Salmonella reduction in commercial broiler flocks. The findings of this investigation identified a link between the H/L ratio and intestinal barrier function and immunity. A low H/L ratio was correlated with an enhanced intestinal barrier and immune response, resulting probably from increased intestinal T-cells, which have been reported to protect the host from bacterial invasion by direct killing or stimulation of the production of several cytokines [85]. According to our enumeration of monocyte, heterophil, and lymphocyte cells, and H/L determination, a low H/L could result from decreased peripheral blood heterophil cells. Therefore, future research focusing on the effects of a low and high H/L ratio on the cecal metabolites (short chain fatty acids) and the microbiota composition of SE-infected chickens will expand our understanding of the mechanisms of Salmonella resistance. Moreover, future experiments should include metagenomic, transcriptomic, and weighted gene co-expression network analysis (WGCNA) data from artificial Salmonella infection at earlier and later stages post-infection as displayed in this study to identify differential metabolites, microbiota composition, and functional capacity, but also differentially expressed genes, immune pathways, and hub genes that are associated with Salmonella-resistance ability.

## 5. Conclusions

The H/L ratio was negatively correlated with IL-1β and IFN-γ ileal expression and the number of goblet cells in the ileum. Taken together, our study revealed that a low H/L ratio is associated with enhanced histomorphometric parameters and with increased expression of immune genes that play key roles in intestinal integrity and Salmonella clearance. Thus, a low H/L ratio could be an immunological status that improves chickens’ intestinal integrity, prevents the loss of goblet cells and mucosal damage, and improves the immune response to Salmonella infection. For these reasons, the H/L ratio could be used as a biomarker to select Salmonella-resistant chickens. In addition, these findings expand our current knowledge on the use of the H/L ratio for research or breeding purposes.

## Figures and Tables

**Table 1:** Descriptive statistics of cells counted and the H/L ratio.

Traits	Mean ± SEM	SD	Min	Max	CV (%)
Monocytes (M)	8.46 ± 0.45	6.49	1	39	76.68
Heterophils (H)	18.2 ± 0.54	7.77	3	47	42.69
Lymphocytes (L)	73.35 ± 0.67	9.56	40	90	13.03
H/L ratio	0.26 ± 0.01	0.16	0.035	1.04	50.04

Mean = arithmetic mean; SEM = standard error of the mean; SD = standard deviation; Min = minimum; Max = maximum; CV = coefficient of variation; 203 out of the 210 chickens were used to generate the presented data.

**Table 2:** Primers.

Genes	Forward (F) and Reverse (R) Primers 5′ to 3′	Accession no./Reference
IL-1β	F: GCATCAAGGGCTACAAGCTCT	[26]
	R: CCAGGCGGTAGAAGATGAAG	
IL-8	F: TCCTCCTGGTTTCAGCTGCT	[26]
	R: GTGGATGAACTTAGAATGAGTG	
IFN-γ	F: CAAGTCAAAGCCGCACATC	[27]
	R: CGCTGGATTCTCAAGTCGTT	
IL-6	F: AATCCCTCCTCGCCAATCT	NM_204628.1
	R: TCACGGTCTTCTCCATAAACG	
LITAF	F: TGTGTATGTGCAGCAACCCGTAGT	[9]
	R: GGCATTGCAATTTGGACAGAAGT	
SOCS3	F: TGCGCCTCAAGACGTTCA	NM_204600.1
	R: GTACTCGCTCTTAGAGCT	
Muc2	F: ACTCCTCCTTTGTATGCGTGA	NM_001318434.1
	R: GTTAACGCTGCATTCAACCTT	
Claudin-1	F: GGTGTACGACTCGCTGCTTA	NM-001013611.2
	R: CGAGCCACTCTGTTGCCATA	
NF-κB	F: CAATGGACCAGCTCATGGGAAT	NM_205134.1
	R: CTTCGCATACGTATCGGAATCG	
TLR4	F: ACGGCATTTCAGAACGGACT	NM_001030693.1
	R: ACAGCTTCTCAGCAGGCAAT	
GAPDH	F: GGAGAAACCAGCCAAGTAT	NM_204305.1
	R: CCATTGAAGTCACAGGAGA	

**Table 3:** Association of H/L with the goblet cells number in the ileum and caecum of non-infected and SE-infected chickens at 7 and 21-days post-infection.

Gut- Segment	Items	Age ^1^	Non-Infected				SE-Infected			
			H/L	GCs (n)	Pearson r	*p*-Values	H/L	GCs (n)	Pearson r	*p*-Values
**Ileum**	Villus	14	0.21	183	−0.5	0.154	0.38	136.3	0.6	0.077
		28	0.18	191.7	−0.78 *	0.019	0.3	173	0.06	0.452
	Crypt	14	0.21	40.25	0.42	0.206	0.38	31.02	−0.35	0.222
		28	0.18	34.43	−0.81 *	0.013	0.3	30.42	−0.64	0.085
**Caecum**	Fold	14	0.24	210.9	−0.84	0.081	0.39	150.5	−0.38	0.232
		28	0.17	129	−0.27	0.300	0.32	88	0.35	0.249
	Crypt	14	0.24	21.93	0.38	0.266	0.39	15.43	−0.41	0.183
		28	0.17	13.46	−0.28	0.272	0.32	8.65	0.08	0.423

^1^ 14 days old correspond to 7 dpi and 28 days old correspond to 21 dpi for SE-infected chickens. GCs (n) = goblet cells number * *p* < 0.05.

**Table 4:** Effects of *Salmonella enteritidis* infection on the ileal villi morphometry at 7 and 21-days post-infection.

Age ^1^	Items	Non-Infected	SE-Infected	*p*-Values
14	VH (µm)	585.82 ± 43.26	512.35 ± 42.20	0.013
	CD (µm)	110.69 ± 36.4	89.79 ± 8.09	0.121
	VW (µm)	95.32 ± 9.44	84.29 ± 7.16	0.035
	VSA (µm^3^)	173,774.42 ± 20,247.01	135,183.81 ± 13,676.20	0.004
	VH/CD ratio	5.82 ± 1.76	5.92 ± 0.57	0.453
	EPT (µm)	32.75 ± 3.71	27.33 ± 3.26	0.020
	LPT (µm)	29.05 ± 4.91	30.22 ± 6.68	0.380
28	VH (µm)	703.75 ± 61.19	554.42 ± 53.99	0.002
	CD (µm)	77.22 ± 15.54	72.95 ± 0.19	0.278
	VW (µm)	129.33 ± 13.14	147.83 ± 27.76	0.107
	VSA (µm^3^)	285,496.87 ± 27,153.06	253,325.51 ± 29,288.02	0.055
	VH/CD ratio	9.62 ± 1.57	7.74 ± 0.80	0.022
	EPT (µm)	46.26 ± 5.18	49.82 ± 10.92	0.265
	LPT (µm)	27.18 ± 2.93	41.97 ± 13.84	0.024

^1^ 14 days old correspond to 7 dpi and 28 days old correspond to 21 dpi for SE-infected chickens. Comparisons between non-infected and SE-infected were performed using Student’s *t*-test (one tail).

**Table 5:** Association of the H/L ratio with the intestinal immune barrier and response to *Salmonella enteritidis* infection at 7 and 21-days post-infection.

Genes	Gut- Segment	14 Days-Old ^1^				28 Days-Old ^2^			
		Non-Infected		SE-Infected		Non-Infected		SE-Infected	
		Pearson r	*p*-Values	Pearson r	*p*-Values	Pearson r	*p*-Values	Pearson r	*p*-Values
IL-1β	Ileum	−0.42	0.113	−0.62 *	0.028	0.3	0.201	0.68 *	0.015
	Caecum	−0.43	0.110	−0.19	0.282	−0.49	0.055	−0.17	0.334
IL-8	Ileum	−0.12	0.367	−0.58 *	0.039	−0.35	0.161	0.70 *	0.012
	Caecum	−0.4	0.100	−0.41	0.095	−0.25	0.216	−0.04	0.464
IFN-γ	Ileum	0.38	0.142	−0.67 *	0.017	−0.19	0.304	0.34	0.172
	Caecum	0.24	0.273	0.27	0.260	−0.48	0.056	0.37	0.164
IL-6	Ileum	−0.3	0.198	−0.51	0.067	0.63 *	0.026	0.64 *	0.024
	Caecum	−0.51 *	0.044	−0.38	0.111	−0.47	0.060	0.47	0.100
LITAF	Ileum	0.58 *	0.041	−0.41	0.120	−0.80 **	0.004	0.07	0.425
	Caecum	0.27	0.200	0.17	0.301	0.15	0.316	−0.41	0.138
SOCS3	Ileum	0.23	0.294	−0.65 *	0.021	−0.43	0.107	0.22	0.268
	Caecum	−0.32	0.154	−0.37	0.117	−0.68 **	0.007	0.25	0.255
MUC2	Ileum	0.36	0.152	−0.44	0.102	−0.42	0.116	−0.5	0.072
	Caecum	−0.63 *	0.014	0.1	0.391	−0.32	0.158	0.26	0.256
Claudin1	Ileum	0.02	0.482	−0.27	0.228	−0.17	0.321	−0.23	0.261
	Caecum	0.38	0.159	−0.05	0.464	−0.44	0.078	0.18	0.321
NF-κB	Ileum	−0.44	0.102	−0.22	0.268	−0.27	0.224	−0.43	0.106
	Caecum	−0.59 *	0.022	0.15	0.318	−0.16	0.310	0.36	0.168
TLR4	Ileum	−0.44	0.102	−0.5	0.071	0.22	0.272	0.15	0.341
	Caecum	−0.38	0.113	−0.04	0.451	−0.44	0.079	0.23	0.276

^1^ 14 days old correspond to 7 dpi. ^2^ 28 days old correspond to 21 dpi. * *p* < 0.05, ** *p* < 0.01.
